# Childhood Growth, IQ and Education as Predictors of White Blood Cell Telomere Length at Age 49–51 Years: The Newcastle Thousand Families Study

**DOI:** 10.1371/journal.pone.0040116

**Published:** 2012-07-06

**Authors:** Mark S. Pearce, Kay D. Mann, Carmen Martin-Ruiz, Louise Parker, Martin White, Thomas von Zglinicki, Jean Adams

**Affiliations:** 1 Institute of Health & Society, Newcastle University, Newcastle Upon Tyne, England, United Kingdom; 2 Institute for Ageing and Health, Newcastle University, Newcastle Upon Tyne, England, United Kingdom; 3 Departments of Medicine and Paediatrics, Dalhousie University, Halifax, Nova Scotia, Canada; INSERM UMR S_910, France

## Abstract

**Background:**

Telomere length is emerging as a potential factor in the pathogenesis of cardiovascular disease. We investigated whether birth weight, infant growth, childhood cognition and adult height, as well as a range of lifestyle, socio-economic and educational factors, were associated with white blood cell telomere length at age 49–51 years.

**Methods:**

The study included 318 members of the Newcastle Thousand Families Study, a prospectively followed birth cohort which includes all individuals born in Newcastle, England in May and June 1947, who attended for clinical examination at age 49–51 years, and had telomere length successfully measured using real-time PCR analyses of DNA extracted from peripheral blood mononuclear cells.

**Results:**

No association was found between birth weight and later telomere length. However, associations were seen with other factors from early life. Education level was the only predictor in males, while telomere length in females was associated with gestational age at birth, childhood growth and childhood IQ.

**Conclusions:**

While these findings may be due to chance, in particular where differing associations were seen between males and females, they do provide evidence of early life associations with telomere length much later in life. Our findings of sex differences in the education association may reflect the sex differences in achieved education levels in this generation where few women went to university regardless of their intelligence. Our findings do not support the concept of telomere length being on the pathway between very early growth and later disease risk.

## Introduction

Telomere length has been reported to be associated with longevity and risk of a number of age-related diseases, including associations with cardiovascular disease [1]–[3], type 2 diabetes mellitus [4], vascular [5] and Alzheimer’s dementia [6]–[7] and solid tissue tumours [8]. However, it has been suggested that the associations with morbidity and mortality are not apparent in very old age [9].

Risk of adverse health in middle age has been proposed to be ‘programmed’ by impaired development in utero [10]. Early growth has also been linked to physical functioning on older age [11]. Telomere length is known to vary at birth [12] and has been suggested to be a biological marker on the pathway between early growth and later health [13]. Further, it has been associated with age-related mortality and morbidity [5]. Low birth weight children may have shorter telomeres in childhood [14], although very low birth weight newborns were found to have longer telomeres in cord blood than low birth weight newborns [15]. However, when comparing small-for-gestational-age newborns to appropriately grown controls, telomere lengths were shown to be similar [16]. Associations between early growth and telomere length in later life do not appear to have been studied previously.

A recent study has suggested a link between education and telomere length in adulthood [17]. However, in addition to studying whether early growth or education are associated with a later outcome such as telomere length, it is also important to address whether factors later in life such as smoking, diet, physical activity and alcohol consumption, may have a more important role in influencing telomere length. It is only by studying detailed longitudinal birth cohort data in an appropriate manner, that questions regarding relative contributions and mediating pathways can be addressed.

The Newcastle Thousand Families birth cohort [18] provides such an opportunity to investigate the determinants of telomere length at age 49–51 years from across the life course and their relative contribution to explaining variation in telomere length. A previous investigation of the telomere data for this cohort found that telomere length was longer in men than in women [19], but did not find associations with socio-economic status or a number of lifestyle factors. The current study investigated the potential associations and interactions between sex and a number of markers of body size at different stages of life, including achieved adult height, childhood cognition, education and further adult lifestyle factors and peripheral blood telomere length at age 49–51 years in members of the Newcastle Thousand Families birth cohort.

## Methods

### Ethics Statement

The study received ethical approval from the South Durham Lead Research Ethics Committee and the Joint Newcastle Health Authority/University of Newcastle upon Tyne Ethics Committee, and all study members gave their written informed consent.

### Study Participants

The Newcastle Thousand Families study began as a prospective study of all 1142 children born in May and June 1947 to mothers resident in Newcastle upon Tyne, UK [18]. The health, growth and development of the cohort were followed in great detail up to age 15. Throughout the first years of the children’s lives, all families were visited both on a routine (up to every six weeks during infancy and at least quarterly until age five years) and on an ad hoc basis by the study team, which consisted of health visitors (nurses who visited families at home) and paediatricians.

The cohort underwent a major follow-up at age 49–51 years [18]. Participants were members of the cohort who were either traced through the National Health Service Central Register or contacted the study team in response to media publicity. Between October 1996 and December 1998, health and lifestyle questionnaires were sent out for completion and return and study members were invited to attend for clinical examination which took place over the same time period.

### Clinical Assessment

Between October 1996 and December 1998, when study members were aged between 49 and 51 years, height, weight and other markers of size were measured. Waist and hip circumferences were measured according to the protocol of the World Health Organisation Monitoring Trends and Determinants in Cardiovascular Disease project [20]. Percent body fat was estimated from impedance measured using a Holtain body composition analyser (Holtain Ltd, Crymych, Wales, UK).

Telomere length in peripheral blood mononuclear cells was measured using real-time polymerase chain reaction (PCR) analysis [21] on DNA extracted from blood donated at age 49–51 years with the following modifications: Measurements were performed in quadruplicate. All PCRs were carried out on an Applied Biosystems 7900HT Fast Real Time PCR machine with 384-well plate capacity. For T/S ratios, the coefficients of variation (CV) were 4.5% (intra-assay) and 6.2% (inter-assay), respectively. In addition, four internal control DNA samples were run within each plate to correct for plate–to-plate variation. This enabled the calculation of absolute telomere length (in kb) from the T/S ratios and further reduced the inter-assay CV to below 5%. The reproducibility of the whole technique including DNA isolation had also been tested in the same laboratory using parallel but independent blood samples [9], resulting in a CV of 2%.

### Measurement of Size and Growth in Early Life

Birth weights, as recorded by the midwife, were standardised for gestational age (as recorded in ante-natal records) and sex [22]. Growth in early childhood was defined as the standard deviation (z) score for height at age nine years, the age in childhood for which the most complete data were available, less the z-score for weight at birth. Childhood BMI, also measured at this age was also included in analyses. Liaison with schools enabled the prospective collection of information on educational performance. In 1958, study members took the 11-plus examination, consisting of written papers involving tests of verbal reasoning (Moray House tests 57 and 58) and two standardized tests of English and arithmetical ability. The total IQ score was derived as the average of the four test results. At that time in England, the 11-plus examination was a standard test used in educational establishments at the age of 11 years, often to determine the type of secondary school at which a child was to continue their education. Results of 11-plus examinations were not available for those children who had moved away from the study area.

### Measurement of Adult Lifestyle

The number of pack-years of cigarettes smoked, current smoking status, physical activity, alcohol consumption and achieved education level were derived from the responses to the self-completion questionnaire data at age 49–51 years [23]. Four categories of alcohol consumption were derived: No drinking; light drinking (up to ten units/week of alcohol for males, 5 units for females); moderate drinking (11–28 units for males, 6–21 units for females) and heavy drinking (>28 units for males, >21 units for females). In the UK, one unit is 10 ml or 8 g of pure alcohol. The number of pack-years of cigarettes smoked (one pack-year  =  one pack of cigarettes smoked per day for one year) was estimated from the study members’ smoking habits at ages 15, 25, 35 and 50, as ascertained at age 49–51 years. Current smoking status (at the time of questionnaire completion) was also derived (never, ex-smoker, current smoker). Physical activity assessment at age 49–51 years was based on that used for the Medical Research Council’s National Survey of Health and Development with four categories (inactive and light, moderate and heavy activity). Achieved education level was classified by the highest achieved qualifications (no qualifications, O-Level (school exit examinations at age 16 years), A-level (school exit examinations at age 18 years), and University degree level and above).

### Statistical Analysis

As the distribution of telomere length was skewed, it was log transformed prior to analyses. Linear regression was used to assess potential associations between log transformed telomere length and potential predictors, and relevant assumptions were tested. Regression coefficients (in log base pairs per unit) and corresponding 95% confidence intervals (95% CI) are reported. Sex-specific analyses and tests for interaction between sex and other potential explanatory variables were done within the linear regression framework. The statistical software package Stata, version 10.0, (StataCorp, College Station: TX) was used for all statistical analyses.

## Results

Of the original 1142 study members, 832 (86% of the surviving sample of 967 children whose families remained in Newcastle for at least the first year of the study) were traced at age 49–51 years ^12^. Of these, 574 completed the health and lifestyle questionnaire and telomere length was measured in 318 study members with available DNA samples (120 men, 198 women). There were no differences in early life factors between the study sample and the remainder of the birth cohort, other than for sex, with more women than men included.

Descriptive statistics for all variables are given in table 1. Mean telomere length was greater in men than in women (p<0.001). Univariate analyses showed strong associations between telomere length and achieved adult height, waist:hip ratio and achieved education at age 49–51 years (table 2). There was little evidence of an association between any early life factors including standardised or crude birth weight and telomere length (p = 0.92 and 0.66 respectively).

**Table 1:** Continuous descriptive statistics by sex.

	Total					Male				Female				
Variable	N		Mean (SD)			N	Mean (SD)			N			Mean (SD)	
Standardised birth weight (Z score)	318		−0.14		(1.09)	120	−0.39		(0.99)	198			0.01	(1.13)
Birth weight (kg)	318		3.37		(0.51)	120	3.35		(0.48)	198			3.38	(0.52)
Childhood growth (change in z-scores: birth and 9 years)	275		−0.01		(0.49)	106	−0.02		(0.49)	169			0.00	(0.50)
Childhood BMI (age 9 years)	269		16.4		(1.99)	103	16.4		(1.92)	166			16.4	(2.04)
Childhood IQ (age 11 years)	266		101.6		(13.9)	103	101.8		(14.3)	163			101.5	(13.7)
Height at age 49–51 years (cm)	314		166.1		(8.44)	118	173.4		(6.66)	196			161.7	(5.94)
BMI at age 49–51 years (kg)	317		26.5		(4.69)	120	26.79		(3.54)	197			26.3	(5.26)
Waist : Hip ratio	316		0.86		(0.10)	120	0.95		(0.06)	196			0.80	(0.06)
Percent body fat	313		39.8		(8.81)	119	36.82		(7.11)	194			41.58	(9.27)
Telomere length at age 49–51 years (log base pairs)	318		8.50		(0.22)	120	8.59		(0.22)	198			8.44	(0.20)
	**N**		**Median (IQR)**			**N**	**Median (IQR)**			**N**			**Median (IQR)**	
Gestational Age (weeks)	317		40	(40,40)		119	40		(40,40)	198			40	(40,40)
Duration Breast Fed (days)	313		61	(23,219)		119	61		(28,223)	194			61.5	(21,219)
Pack years cigarettes	316		2.25	(0,22.1)		119	8.3		(0,29.59)	197			0.7	(0,17.65)
Telomere length at age 49–51 years (base pairs)	318		5016	(1151)		120	5512		(1210)	198			4716	(1003)
	**N**		**%**			**N**	**%**			**N**			**%**	
Sex	318		100			120	38			198			62	
Social class at birth														
I,II	32	10				14		12			18		9	
III	200	64				70		60			130		66	
IV,V	81	26				33		28			48		25	
Smoking status age 49–51 years														
Never Smoked	137	42				41		34			96		48	
Ex Smoker	92	29				47		40			45		22	
Current Smoker	87	28				31		26			56		28	
Alcohol consumption at age 49–51 years (self reported)														
None	38	12				10		8			28		14	
Light	126	40				49		41			77		39	
Medium	126	40				47		40			79		41	
Heavy	24	8				13		11			11		6	
Social class at age 49–51 years														
I,II	156	52				62		54			94	51		
III	100	34				39		34			61	33		
IV,V	42	14				13		12			29	16		
Physical activity at age 49–51 years														
Inactive	32	10				11		10			21	11		
Light Activity	150	50				61		54			89	48		
Moderate Activity	68	23				23		20			45	24		
Heavy Activity	50	17				18		16			32	17		
Achieved education level at age 49–51 years														
No Qualifications	104	34				30		26			74	39		
O Level or equivalent	105	34				35		30			70	37		
A level or equivalent	58	19				32		28			26	13		
Degree/post graduate	39	13				18		16			21	11		

**Table 2:** Univariate associations between telomere length (log base pairs) and explanatory variables.

		All		All, adjusted for sex		Male		Female	
Variable		β (95% CI)	p	β (95% CI)	P	β (95% CI)	p	β (95% CI)	p
Sex (male reference category)		−0.15(−0.20, −0.11)	<0.001*	−	–	–	–	–	–
Standardised birth weight		−0.01 (−0.02,0.02)	0.92	0.01 (−0.01,0.03)	0.29	0.02 (−0.02,0.06)	0.30	0.01 (−0.02,0.03)	0.59
Birth weight (kg)		0.01 (−0.04,0.06)	0.66	0.01 (−0.03,0.06)	0.53	0.06 (−0.02,0.15)	0.12	−0.01 (−0.06,0.04)	0.69
Gestational Age (weeks)		−0.01 (−0.03,0.01)	0.49	−0.02 (−0.03,0.01)	0.31	0.02 (−0.02,0.06)	0.25	−0.03 (−0.05, −0.03)	0.03*
Duration Breast Fed (weeks)		−0.01 (0.00, 0.01)	0.46	−0.00 (0.00, 0.01)	0.45	0.00 (0.00,0.01)	0.26	0.00 (0.00, 0.00)	0.99
Social class at birth	I,II	−0.01 (−0.09,0.08)	0.82	−0.02 (−0.10,0.06)	0.81	−0.05 (−0.18,0.08)	0.74	0.00 (−0.09,0.09)	0.84
	III	reference		reference		reference		reference	
	IV,V	0.02 (−0.04,0.07)		0.01 (−0.05,0.06)		−0.01 (−0.01,0.08)		0.02 (−0.05,0.09)	
Childhood growth		0.02 (−0.03,0.07)	0.39	0.03 (−0.02,0.08)	0.31	−0.03 (−0.12,0.06)	0.53	0.06 (−0.01,0.12)	0.05
Childhood IQ (age 11)		−0.01 (−0.03,0.00)	0.13	0.00 (−0.01,0.02)	0.09	0.00 (−0.03,0.03)	0.99	−0.02 (−0.04, −0.01)	0.02*
Height at age 49–51 years (cm)		0.01 (0.01,0.01)	0.002*	0.00 (−0.01,0.01)	0.28	−0.01 (−0.01,0.01)	0.63	−0.02 (−0.01,0.02)	0.30
BMI at age 49–51 years		0.01(−0.01,0.01)	0.67	0.00 (−0.01,0.01)	0.88	0.01 (−0.01,0.01)	0.84	0.00 (−0.01,0.01)	0.95
Waist : Hip ratio		0.57 (0.32,0.82)	<0.001*	−0.13 (−0.53,0.27)	0.53	−0.33 (−1.04,0.37)	0.35	−0.01 (−0.49,0.47)	0.97
Percent body fat		−0.01 (−0.01,0.01)	0.26	0.00 (−0.01,0.00)	0.67	0.01 (−0.01,0.01)	0.86	0.01 (−0.02,0.03)	0.69
Pack years cigarettes		0.01 (0.00,0.01)	0.35	−0.01 (−0.02,0.01)	0.67	0.00 (−0.01,0.01)	0.48	0.00 (−0.02,0.03)	0.82
\Smoking statusage 49–51 years	Never Smoked	reference	0.39	reference	0.03*	reference	0.18	reference	0.17
	Ex Smoker	−0.03 (−0.09,0.02)		−0.07 (−0.13, −0.01)		−0.08 (−0.18,0.01)		−0.06 (−0.13,0.01)	
	Current Smoker	0.01 (−0.05,0.07)		−0.02 (−0.06,0.05)		−0.02 (−0.13,0.08)		0.01 (−0.05,0.07)	
Alcohol consumptionat age 49–51 years	None	reference	0.98	reference	0.87	reference	0.72	reference	0.45
	Light	−0.02 (−0.10,0.07)		0.01 (−0.07,0.08)		−0.08 (−0.23,0.07)		0.03 (−0.05,0.12)	
	Medium	−0.01(−0.06,0.05)		−0.01 (−0.06,0.05)		0.01 (−0.08,0.10)		−0.01 (−0.08,0.05)	
	Heavy	−0.01(−0.11,0.08)		−0.04 (−0.13,0.06)		−0.01 (−0.13,0.14)		−0.08 (−0.21,0.05)	
Social class at age49–51 years	I,II	−0.01 (−0.06,0.05)	0.96	−0.01 (−0.06,0.05)	0.98	0.04 (−0.05,0.13)	0.66	−0.03 (−0.10,0.03)	0.61
	III	reference		reference		reference		reference	
	IV,V	−0.01 (−0.09,0.07)		0.01 (−0.07,0.08)		0.04 (−0.11, 0.18)		−0.02 (−0.11,0.07)	
Physical activity atage 49–51 years	Inactive	reference	0.92	reference	0.91	reference	0.10	reference	0.24
	Light Activity	0.02(−0.07,0.10)		0.01 (−0.07,0.09)		−0.12 (−0.26,0.02)		0.08 (−0.01,0.18)	
	Moderate	0.01(−0.08,0.1)		0.01 (−0.08,0.10)		−0.02 (−0.17,0.14)		0.03 (−0.08,0.13)	
	Heavy Activity	0.03 (−0.07,0.05)		0.03 (−0.02, −0.11)		−0.04 (−0.21,0.12)		0.07 (−0.4,0.18)	
Achieved education level atage 49–51 years	No Qualifications	reference	0.04*	reference	0.01*	reference	0.01*	reference	0.16
	O Level or equivalent	−0.08 (−0.13, −0.02)		−0.08 (−0.14, −0.03)		−0.12 (−0.22, −0.02)		−0.07 (−0.13,0.00)	
	A level or equivalent	−0.04 (−0.11,0.03)		−0.08 (−0.15, −0.01)		−0.11 (−0.21, −0.03)		−0.05 (−0.14,0.04)	
	Degree/post graduate	0.01(−0.07,0.09)		−0.01 (−0.09, −0.06)		0.06 (−0.06,0.18)		−0.08 (−0.18,0.01)	

After adjusting for sex, neither achieved adult height or waist:hip ratio remained associated with telomere length (p = 0.28 and 0.53 respectively). However, the negative association between achieved education level and telomere length remained (p = 0.01). In sex-specific analyses, there were associations between telomere length and gestational age (p = 0.03), childhood growth (p = 0.05) and childhood IQ (p = 0.02) in women. Achieved education level at age 49–51 years in men was associated with telomere length (p<0.01). When compared to the reference category of no qualifications, lesser telomere lengths were seen for those with O and A-levels, but slightly longer telomeres were seen in those with university qualifications. Interactions were seen between sex and both gestational age (p = 0.026) and achieved education level (p = 0.05) on telomere length. Increasing male gestational age was associated with increased telomere length in contrast to an association between decreased length and increasing female gestation. There were decreasing telomere lengths for higher educational achievement in females, but with the longest telomere lengths in the highest achieving males. There was no evidence of interactions between sex and any of the other explanatory variables on telomere length.

Of the associations observed, the highest explanation of variation in the data was seen for sex (r^2^ = 0.12). Within sex-specific analyses, male achieved education level and female childhood IQ and gestational age accounted for 11%, 4% and 2.4%, respectively, of the variation in log transformed telomere length. After adjustment for sex, the resulting model including education and interactions was found to explain 15% of the variation in log transformed telomere length.

## Discussion

### Principal Findings

While associations were seen between telomere length and both achieved adult height and contemporary waist:hip ratio, neither association was independent of sex. This is likely to be due to men being both taller and having greater waist:hip ratios and having longer telomeres in this cohort. An association was seen between achieved education level and telomere length, with an interaction with sex. Achieved education level was the only association with telomere length in men, while gestational age, childhood IQ and childhood growth (from birth to age nine years) were associated in women.

### Strengths and Potential Weaknesses

The main strength of this study is the ability to analyse prospectively collected data from different stages of life simultaneously. Of 1142 men and women recruited at birth in 1947, 28% participated in the current study. Except for sex, the study sample attending for clinical examination has been shown to be comparable for a wide range of explanatory variables in early life ^23^. In addition, inclusion of cohort members who had moved out of the study region (18% of those who attended for clinical examination were resident outside the Northern Region of England) increased the representativeness of the population studied. However, the potential for participation bias in terms of later factors remains a possibility and the study may have been underpowered for some variables, particularly for the sex-specific analyses. Despite the small numbers in these analyses, though, a number of associations and interactions were identified and the final model accounted for 15% of the variation in telomere length.

Telomere length is known to vary with age. All cohort members were born within a two-month period and assessed when aged between 49 and 51 years, reducing the potential for bias. Furthermore, they were all born to mothers resident within the city of Newcastle upon Tyne in the north east of England, so should have less genetic and environmental heterogeneity than would be found in a study incorporating a larger geographical area, or one with ethnic diversity [24].

### Comparisons with Other Studies

We have previously reported that the men in this cohort had, on average, longer telomeres than the women at age 49–51 years [19]. This is contrast to the majority of other studies reporting longer telomeres in women or no difference between men and women [24]–[28]However, a recent study of Scottish 70-year-olds also found longer telomeres in males when compared to females [29].We are not aware of any cohort-specific behavioural patterns or environmental exposures that could explain this observation, although in the older Scottish cohort it is possible that factors such as the selective survival of men with longer telomeres may play a role.

There were associations between both achieved adult height and contemporary waist:hip ratio and telomere length at age 49–51years in unadjusted analyses, but neither association remained after adjustment for sex or in sex-specific analyses. For waist:hip ratio, this is likely to reflect the larger waist:hip ratio in men compared to women.

Although telomere length is suggested to vary between men and women, such a difference is not apparent at birth, despite the variability in telomere length among newborns [12]. Very low birth weight newborns have been found to have longer telomeres in cord blood than low birth weight newborns [15], while fetal growth restriction has been associated with reduced telomere length [30]. Low birth weight children have been found to have shorter telomeres in childhood [14], but do not appear to have been studied in terms of telomere length later in life. Telomere length at age 49–51 years and both crude and standardised birth weight were slightly greater in males than in females, though not to the extent that they could be included in the adjusted model. Gestational age was inversely associated with telomere length in women. This, to our knowledge, has not previously been reported and requires replication in other cohorts to rule out the possibility of it being a chance finding, particularly given the number of potential predictor variables included in this analysis.

Our finding of an association between childhood growth and telomere length at age 49–51 years was restricted to women, with a higher change in z-score between birth and height at age nine years associated with longer telomeres. Obesity in childhood has been reported to be associated with shorter telomere length, again measured in childhood [31], but does not appear to have been studied in relation to telomere length later in life.

Childhood IQ (in females) and achieved education level (in males) were both associated with telomere length. The association with educational attainment for men, in that the longest telomeres were seen in those with the highest achieved education level, is consistent with recent evidence reporting that the associations with telomere length were dependent on educational attainment rather than contemporary socio-economic circumstances [17], [32]. Also consistent with this is our previous finding of no association with socio-economic status [19]. Telomere length has been inconsistently associated with contemporary cognitive function [29], [33]–[34], although not with cognitive decline [35]. That education was the significant factor in men while it was childhood IQ in women may be explained by the social values of the time, where education was not considered as important for women. Members of this cohort were teenagers in the 1960 s when only a small percentage of the population went to university and there were still high levels of inequality between the sexes, with less opportunity for women to achieve a high education level even with a relatively high IQ [36]. However, the lack of consistent results in the expected directions for both males and females may also suggest that some of the significant findings in the sex-specific analyses may be due to chance or due to residual confounding.

### Conclusion

Our findings suggest that, while achieved adult height and waist:hip ratio at age 49–51 years appear to be associated with telomere length at the same age, this is likely to reflect the differences in these measures between men and women and thus be due to confounding. No association was found between birth weight and later telomere length. However, significant associations were seen with other factors from early life. Education level was the only predictor in males, possibly reflecting the higher education levels in males in this generation, while telomere length in females was associated with gestational age, childhood growth and childhood IQ. While these findings may be due to chance, in particular where differing associations were seen between males and females, they do provide evidence of early life associations with telomere length much later in life. However, they do not support the concept of telomere length being on the pathway between very early growth and later disease risk.
